# The impact of regional origin on the incidence of gestational diabetes mellitus in a multiethnic European cohort

**DOI:** 10.3389/fpubh.2023.1286056

**Published:** 2024-01-19

**Authors:** Grammata Kotzaeridi, Cécile Monod, Tina Linder, Daniel Eppel, Vera Seidel, Michael Feichtinger, Beatrice Mosimann, Valeria Filippi, Silke Wegener, Wolfgang Henrich, Andrea Tura, Christian S. Göbl

**Affiliations:** ^1^Department of Obstetrics and Gynaecology, Medical University of Vienna, Vienna, Austria; ^2^Department of Obstetrics and Gynaecology, University Hospital Basel, Basel, Switzerland; ^3^Clinic of Obstetrics, Charité-Universitätsmedizin Berlin, Corporate Member of Freie Universität Berlin, Humboldt-Universität zu Berlin, and Berlin Institute of Health, Berlin, Germany; ^4^Wunschbaby Institute Feichtinger, Vienna, Austria; ^5^Metabolic Unit, CNR Institute of Neuroscience, Padova, Italy; ^6^Department of Obstetrics and Gynaecology, Division of Obstetrics, Medical University of Graz, Graz, Austria

**Keywords:** gestational diabetes mellitus, ethnicity, risk prediction, glucose levels, migration, risk stratification

## Abstract

**Introduction:**

Women with migration background present specific challenges related to risk stratification and care of gestational diabetes mellitus (GDM). Therefore, this study aims to investigate the role of ethnic origin on the risk of developing GDM in a multiethnic European cohort.

**Methods:**

Pregnant women were included at a median gestational age of 12.9 weeks and assigned to the geographical regions of origin: Caucasian Europe (*n* = 731), Middle East and North Africa countries (MENA, *n* = 195), Asia (*n* = 127) and Sub-Saharan Africa (SSA, *n* = 48). At the time of recruitment maternal characteristics, glucometabolic parameters and dietary habits were assessed. An oral glucose tolerance test was performed in mid-gestation for GDM diagnosis.

**Results:**

Mothers with Caucasian ancestry were older and had higher blood pressure and an adverse lipoprotein profile as compared to non-Caucasian mothers, whereas non-Caucasian women (especially those from MENA countries) had a higher BMI and were more insulin resistant. Moreover, we found distinct dietary habits. Non-Caucasian mothers, especially those from MENA and Asian countries, had increased incidence of GDM as compared to the Caucasian population (OR 1.87, 95%CI 1.40 to 2.52, *p* < 0.001). Early gestational fasting glucose and insulin sensitivity were consistent risk factors across different ethnic populations, however, pregestational BMI was of particular importance in Asian mothers.

**Discussion:**

Prevalence of GDM was higher among women from MENA and Asian countries, who already showed adverse glucometabolic profiles at early gestation. Fasting glucose and early gestational insulin resistance (as well as higher BMI in women from Asia) were identified as important risk factors in Caucasian and non-Caucasian patients.

## 1 Introduction

In parallel to the rising rates of metabolic disorders that affect the general and progressively more the reproductive-aged younger population, the prevalence of gestational diabetes mellitus (GDM) has increased over the last decades. Nowadays, the global prevalence of GDM ranges between 12 and 18% of pregnancies, with regional prevalence varying from 7% in North America to 27% in MENA countries (1–3). Thereby, hyperglycemia does not only elevate the risk of adverse outcome for women and offspring during pregnancy and at birth, but also the long-term risk of cardiovascular disease and type 2 diabetes (4).

It is known that the GDM prevalence markedly differs between different ethnic populations and hence, the region of origin has been recognized as a non-negligible risk factor. However, detailed data about each area and its specific role and importance are sparse and sometimes conflicting (4–9). Although Asian populations and women from the Sub-Saharan region have long been recognized as being at high risk of developing the disease, women from other regions are also often affected (3, 10, 11). For example, more recent evidence suggests an increased prevalence of GDM in pregnant women from the Middle East and North Africa countries as compared to other populations (3, 12). Migration has been steadily rising and the countries of origin have greatly diversified over the past years. As a consequence, women with migration background were reported to constitute up to 8–14% of the collective of pregnant women in two Nordic countries (8, 13). In this context, pregnant women with migration background (especially those from other continents) present specific challenges related to detection and care of GDM that need to be addressed (14). However, prospective studies, including information of clinical features, metabolic parameters and dietary habits in multiethnic cohorts are sparsely available.

Therefore, this study aims to investigate and refine the possible role of non-Caucasian ancestry for the development of GDM in a multiethnic European cohort. Moreover, ethnic specific differences in glucometabolic parameters and dietary habits at start of pregnancy as well as possible differences in their contribution for GDM development will be assessed in different ethnic groups.

## 2 Methods

### 2.1 Study design and patients

The study design is reported in detail elsewhere (15). In short, pregnant women who participated in this prospective cohort study were recruited among patients attending the pregnancy outpatient clinic at the Department of Obstetrics and Gynaecology, Division of Obstetrics and feto-maternal Medicine, Medical University of Vienna between 2016 and 2019. Patients with preexisting diabetes (such as type 1 or type 2 diabetes) or those with early gestational glycated haemoglobin A1c (HbA1c) equal or exceeding 6.5% at study entry were excluded. Study participants were included at a median gestational age of 12.9 weeks, interquartile range (IQR) 12.3 to 13.6 weeks. An assessment of maternal characteristics (e.g., maternal age, parity, obstetric history, family history of diabetes, as well as women’s height and pregestational weight and body mass index (BMI)) were assessed at time of recruitment. Moreover, we collected detailed information about the country of origin of participating women and their parents. Therefore, we defined five geographical regions: Caucasian (*n* = 731), Middle East and North Africa (MENA, *n* = 195), Asia (*n* = 127), Sub Saharan African (SSA, *n* = 48) and Others and assigned each country present in our cohort to one region according to the definition of the NCD Risk Factors Collaboration (NCD-RisC) (16). We only included women whose parents’ countries of birth were known and situated within the same region and hence excluded 22 women. In the non-Caucasian cohort, about 88.2% of women were first generation migrants and 11.8% were second generation migrants. Table 1 gives an overview of the number of women included and their regions and countries of origin. Thereafter, participants were followed-up until delivery to assess status of GDM and pregnancy outcomes.

**Table 1:** List of countries and study participants included for each region.

Region	Country	*n*	Region	Country	*n*	Region	Country	*n*
**Europe**	Albania	3	**Middle East and North Africa (MENA)**	Algeria	1	**Africa**	Cameroon	1
	Austria	272		Egypt	16		Congo	4
	Belarus	1		Iran	7		Cote d’Ivoire	1
	Bosnia	51		Iraq	7		Ethiopia	1
	Bulgaria	16		Jordan	1		Gambia	1
	Croatia	19		Lebanon	2		Ghana	1
	Czech Republic	9		Syria	32		Nigeria	13
	Denmark	1		Tunisia	2		Somalia	25
	Estonia	1		Turkey	127		Sudan	1
	Finland	1	**Asia**	Afghanistan	52	**Others**	Bolivia	1
	France	1		Armenia	2		Chile	1
	Germany	20		Azerbaijan	1		Ecuador	1
	Greece	2		Bangladesh	8		El Salvador	1
	Hungary	12		China	8		Haiti	1
	Italy	5		India	12		Peru	3
	Kosovo	28		Georgia	4		USA	1
	Latvia	1		Kazakhstan	2			
	Moldavia	1		Kyrgyzstan	1			
	North Macedonia	15		Mongolia	2			
	Poland	27		Pakistan	8			
	Romania	48		Philippines	17			
	Russia	28		Uzbekistan	5			
	Serbia	131		South/North Korea	1			
	Slovakia	25		Sri Lanka	2			
	Spain	5		Thailand	1			
	Switzerland	1		Vietnam	1			
	Ukraine	4						
	United Kingdom	3						

If both parents were born in different countries in the same region, the country of birth of the mother was considered in this table.

### 2.2 Metabolic characterization

At time of recruitment, fasting plasma glucose (FPG), insulin and HbA1c as well as triglycerides, total-cholesterol, LDL-cholesterol and HDL-cholesterol were assessed and the homeostasis model assessment of insulin resistance (HOMA-IR) was evaluated (17). Universal Screening of GDM was routinely performed by use of a 75 g 2 h oral glucose tolerance test (OGTT) in the late second or early third trimester. Thereby, the diagnosis of GDM was based on the cut-offs after oral glucose load proposed by the International Association of the Diabetes and Pregnancy Study Groups (IADPSG) criteria (18). The standard laboratory methods at our certified Department of Medical and Chemical Laboratory Diagnostics^1^ were used to determine all laboratory parameters in this study. According to the international and local guidelines, glucose measurements during the diagnostic OGTT were assessed by use of venous plasma blood samples at local public laboratories (19). Moreover, a food frequency questionnaire was used at time of inclusion to address dietary habits at the start of pregnancy (20).

### 2.3 Ethics approval

The Ethics Committee of the Medical University of Vienna approved the study (EK 1937/2015). The study was performed in accordance with the Declaration of Helsinki. All participants gave written informed consent.

### 2.4 Statistical analysis

Continuous variables were reported as mean ± standard deviation and in case of skewed distributed data as median and interquartile ranges (IQR). These were compared by either Welch’s *t*-test (for two samples) and analysis of variance (for more than two samples), or rank based “nonparametric” inference such as the Kruskal Wallis Test, respectively. Categorical variables were summarized by counts and percentages and compared by binary logistic regression, whereby odds ratios and 95% Confidence Intervals (95%CI) were calculated for dichotomous outcomes (such as the development of GDM) by binary logistic regression. Multiple logistic regression was further used to identify a possible effect of non-Caucasian ethnicity on the risk of GDM adjusted for various confounders. Thereby, stepwise variable selection was used to identify the model with the lowest (i.e., best) Akaike’s information criterion (AIC). For comparison of more than two groups with one reference group we used Dunnett’s *post hoc* test to achieve a 95% coverage probability. We further used recursive partitioning to calculate variable importance metrics as the average difference in predictive accuracy before and after random permutation of the values of a predictor variable over 10^6^ random decision trees (21). Statistical analysis was performed with R (version 4.2.2) and contributing packages (especially “multcomp,” “nparcomp” and “randomForest” as well as “ggplot2” for visualisations) (22, 23). A two-sided *p*-value of ≤0.05 was considered statistically significant. All reported *p*-values were interpreted in an explorative manner aiming to generate new hypotheses.

## 3 Results

### 3.1 Characteristics of women with different ethnicity at early pregnancy

Characteristics of Caucasian and non-Caucasian study participants are provided in Table 2. Pregnant women of Caucasian origin were older, more often nulliparous and used assisted reproduction more frequently. Moreover, Caucasian mothers were more likely to smoke (or to be former smokers) and characterized by higher blood pressure and increased total-cholesterol, LDL-cholesterol but also HDL- and non-HDL-cholesterol as compared to non-Caucasian mothers at the beginning of pregnancy. In contrast, non-Caucasian women were more often multiparous, and showed higher BMI as well as a higher degree of insulin resistance associated with modestly higher HbA1c, fasting glucose and insulin levels. A more detailed comparison of early gestational metabolic parameters in pregnant women according to the regional origin is provided in the Supplementary Table S1, showing distinct differences between the investigated subgroups. Women from the MENA as well as those from the SSA region had higher BMI as compared to Caucasian mothers. Women from MENA countries were more insulin resistant with elevated fasting glucose and insulin levels, while women from SSA and Asia showed higher HbA1c but lower total- and non-HDL-cholesterol as compared to the Caucasian population. Distinct differences were also observed in dietary habits (Table 3). Non-Caucasian mothers consumed more rice, couscous or bulgur and legumes but less noodles, potatoes, vegetables, meat and fruits. However, a more detailed analysis of dietary habits according to the regional origin showed that especially women from the MENA regions consumed more carbohydrates such as bread, rice, couscous or bulgur, but were also eating more legumes as compared to Caucasian mothers. A higher rice, bulgur or couscous consumption was also observed in SSA and Asian mothers, whereas they were eating sweets less often.

**Table 2:** Early gestational characteristics of women with Caucasian and non-Caucasian origin.

	*n*	CAUC	*n*	NON-CAUC	*p*-value
Age (years)	731	32.0 ± 5.9	379	31.2 ± 5.6	0.022
Parity (≥1)	731	404 (55.3)	379	287 (75.7)	<0.001
Parity (≥2)	731	154 (21.1)	379	162 (42.7)	<0.001
Parity (≥3)	731	46 (6.3)	379	80 (21.1)	<0.001
GDM, previous pregnancy	731	67 (9.2)	379	51 (13.5)	0.029
Family history (1st degree)	731	164 (22.4)	379	134 (35.4)	<0.001
Assisted reproduction	731	93 (12.7)	379	28 (7.4)	0.008
Multiple pregnancy	731	95 (13.0)	379	32 (8.4)	0.025
Smoking status (actual smokers)	731	128 (17.5)	379	27 (7.1)	<0.001
Smoking status (former smokers)	731	232 (31.7)	379	41 (10.8)	<0.001
Smoking status (actual and former smokers)	731	360 (49.2)	379	68 (17.9)	<0.001
Pack years (a)	723	0 (0–4)	376	0 (0–0)	<0.001
Height (cm)	731	166 ± 6.6	379	162 ± 6.4	<0.001
Weight, before pregnancy (kg)	731	68 ± 16	379	67 ± 13	0.188
BMI, before pregnancy (kg/m^2^)	731	24.7 ± 5.7	379	25.4 ± 4.9	0.018
RRS (mmHg)	731	120 ± 12	378	117 ± 13	<0.001
RRD (mmHg)	731	77 ± 10	378	75 ± 10	0.010
Triglycerides, early pregnancy (mg/dl)	692	119 ± 47	361	120 ± 47	0.850
Total-cholesterol, early pregnancy (mg/dl)	695	192 ± 35	364	183 ± 34	<0.001
LDL-cholesterol, early pregnancy (mg/dl)	695	96 ± 28	363	92 ± 27	0.008
HDL-cholesterol, early pregnancy (mg/dl)	695	72 ± 16	363	67 ± 15	<0.001
non-HDL-Cholesterol, early pregnancy (mg/dl)	695	120 ± 33	363	115 ± 31	0.026
FPG, early pregnancy (mg/dl)	690	81.2 ± 6.3	362	82.9 ± 7.1	<0.001
HbA1c, early pregnancy (mmol/mol)	700	30.7 ± 3.2	364	31.7 ± 3.5	<0.001
Fasting insulin, early pregnancy (μU/ml)	663	7.7 (5.3–11.6)	350	8.9 (6.3–13.1)	<0.001
HOMA-IR, early pregnancy (dimensionless)	656	1.6 (1.0–2.4)	347	1.8 (1.3–2.7)	<0.001

Data are number of available cases (*n*), mean ± SD or median (IQR) and count (%) for women with Caucasian (CAUC) and non-Caucasian (NON-CAUC) origin. GDM, gestational diabetes mellitus; BMI, body mass index; RRS, systolic blood pressure; RRD, diastolic blood pressure; LDL, low density lipoprotein; HDL, high density lipoprotein; FPG, fasting plasma glucose; HbA1c, glycated haemoglobin A1c; HOMA-IR, homeostasis model assessment of insulin resistance.

**Table 3:** Dietary habits at early pregnancy of women with Caucasian and non-Caucasian origin.

	*n*	CAUC	*n*	NON-CAUC	*p*-value
Milk (ml/d)	627	100 (21–200)	291	43 (9–200)	<0.001
Water (l/d)	627	1.2 (0.9–4.8)	287	1.2 (0.6–3.6)	<0.001
Non-alcoholic beverages (ml/d)	597	200 (61–443)	261	104 (36–300)	<0.001
Coffee (ml/d)	632	32 (0–150)	283	3 (0–32)	<0.001
Tea (ml/d)	618	75 (8.0–182)	278	182 (67–581)	<0.001
Bread (g/d)	603	82 (48–150)	261	100 (50–200)	0.062
Rice, couscous, bulgur (g/d)	616	16 (7–32)	276	32 (13–75)	<0.001
Noodles (g/d)	615	27 (11–27)	275	11 (4–27)	<0.001
Potatoes (g/d)	614	49 (26–84)	259	40 (20–77)	0.003
Pizza (g/d)	617	13 (6–31)	273	13 (3–31)	<0.001
Breakfast cereals (g/d)	614	3 (0–10)	262	0 (0–5)	<0.001
Legumes (g/d)	627	7 (3–16)	276	13 (3–32)	0.005
Vegetables (g/d)	608	88 (34–182)	260	75 (20–150)	0.024
Fruits (g/d)	623	300 (130–450)	272	185 (150–320)	0.133
Butter and magarine (g/d)	626	4 (1–10)	286	1 (0–8)	<0.001
Cheese (g/d)	619	15 (5–30)	281	6 (1–30)	<0.001
Cream Cheese (g/d)	627	3 (1–13)	283	3 (0–15)	0.079
Curd cheese, soured milk, yoghurt (g/d)	617	43 (9–100)	285	43 (9–100)	0.379
Eggs (g/d)	624	13 (5–26)	287	26 (11–60)	<0.001
Meat (g/d)	624	26 (5–26)	283	13 (4–26)	0.013
Meat products (g/d)	616	12 (4–26)	276	0 (0–4)	<0.001
Poultry (g/d)	625	16 (13–32)	284	13 (3–32)	<0.001
Fish (g/d)	612	8 (2–19)	264	3 (0–10)	<0.001
Fast Food (g/d)	614	13 (0–25)	273	10 (0–25)	0.055
Sweat spreads (g/d)	628	3 (1–8)	283	4 (1–10)	0.283
Sweets (g/d)	595	38 (21–72)	257	27 (11–56)	<0.001
Salty snacks (g/d)	613	4 (2–10)	253	4 (2–11)	0.301

Data are number of available cases (*n*), mean ± SD or median (IQR) and count (%) for women with Caucasian (CAUC) and non-Caucasian (NON-CAUC) origin.

### 3.2 Association of ethnicity and the development of GDM

The prevalence of GDM was higher in non-Caucasian mothers as compared to the Caucasian population (*n* = 108, 28.5% vs. *n* = 128, 17.5%, OR 1.87, 95%CI 1.40 to 2.52, *p* < 0.001) and comparable results were observed after adjustment for maternal age and BMI (adjusted OR 1.90, 95%CI 1.41 to 2.58, *p* < 0.001). The results remained unchanged in a fully adjusted logistic regression model including the variables provided in Tables 2, 3 (adjusted OR 2.95, 95%CI 1.32 to 6.60, *p* = 0.008) as well as in a reduced model using stepwise selection (adjusted OR 3.38, 95%CI 1.74 to 6.60, *p* < 0.001). Likewise, non-Caucasian mothers showed higher glucose concentrations within the diagnostic OGTT vs. Caucasian mothers (OGTT glucose baseline: 83 ± 9 vs. 81 ± 10 mg/dL, *p* = 0.001; OGTT glucose 60′: 142 ± 37 vs. 132 ± 33 mg/dL, *p* < 0.001; OGTT glucose 120′: 113 ± 28 vs. 106 ± 25 mg/dL, *p* < 0.001) and required glucose lowering medications more often (*n* = 61, 56.5% vs. *n* = 53, 41.4%, *p* = 0.022 for non-Caucasian vs. Caucasian GDM patients, respectively). A detailed analysis of regional origin showed that the increased incidence of GDM was especially observed in MENA (OR 1.95, 95%CI 1.35 to 2.79, *p* < 0.001) and Asian mothers (OR 2.09, 95%CI 1.36 to 3.17, *p* < 0.001). However, no differences were observed in pregnancy outcomes including cesarean section rate (*p* = 0.919), international birth weight percentiles (*p* = 0.980) or the incidence of LGA offspring (*p* = 0.871) and the results remained unchanged when women with normal glucose tolerance were excluded. Likewise, we found no significant difference in LGA delivery in previous pregnancy.

### 3.3 Risk factors for GDM stratified by ethnic origin

**Figure 1:**
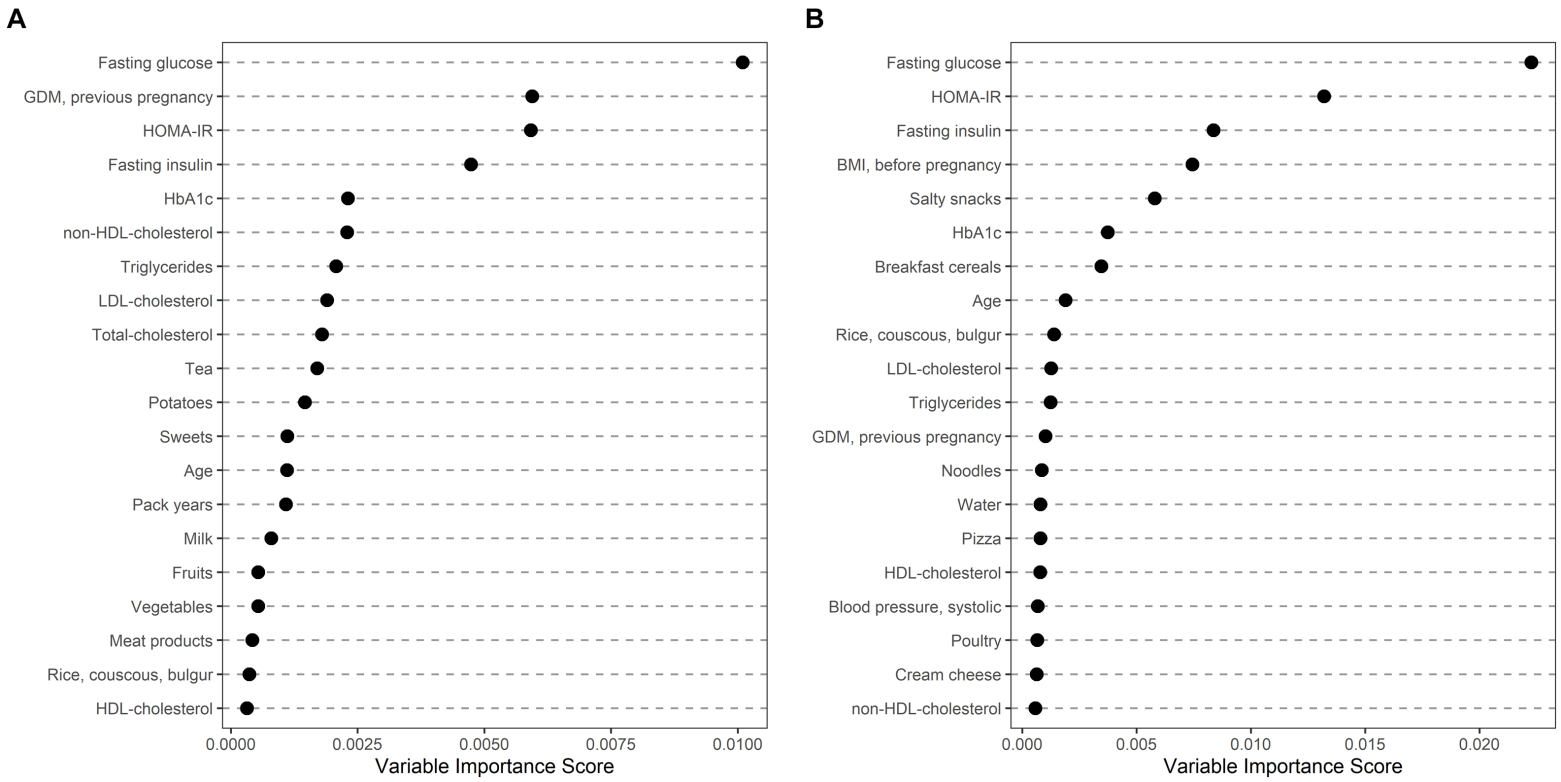
Ethnically stratified risk factors for GDM at early pregnancy showing variable importance scores for mothers with Caucasian **(A)** and non-Caucasian **(B)** origin. The first 20 variables with highest variable scores are shown.

**Figure 2:**
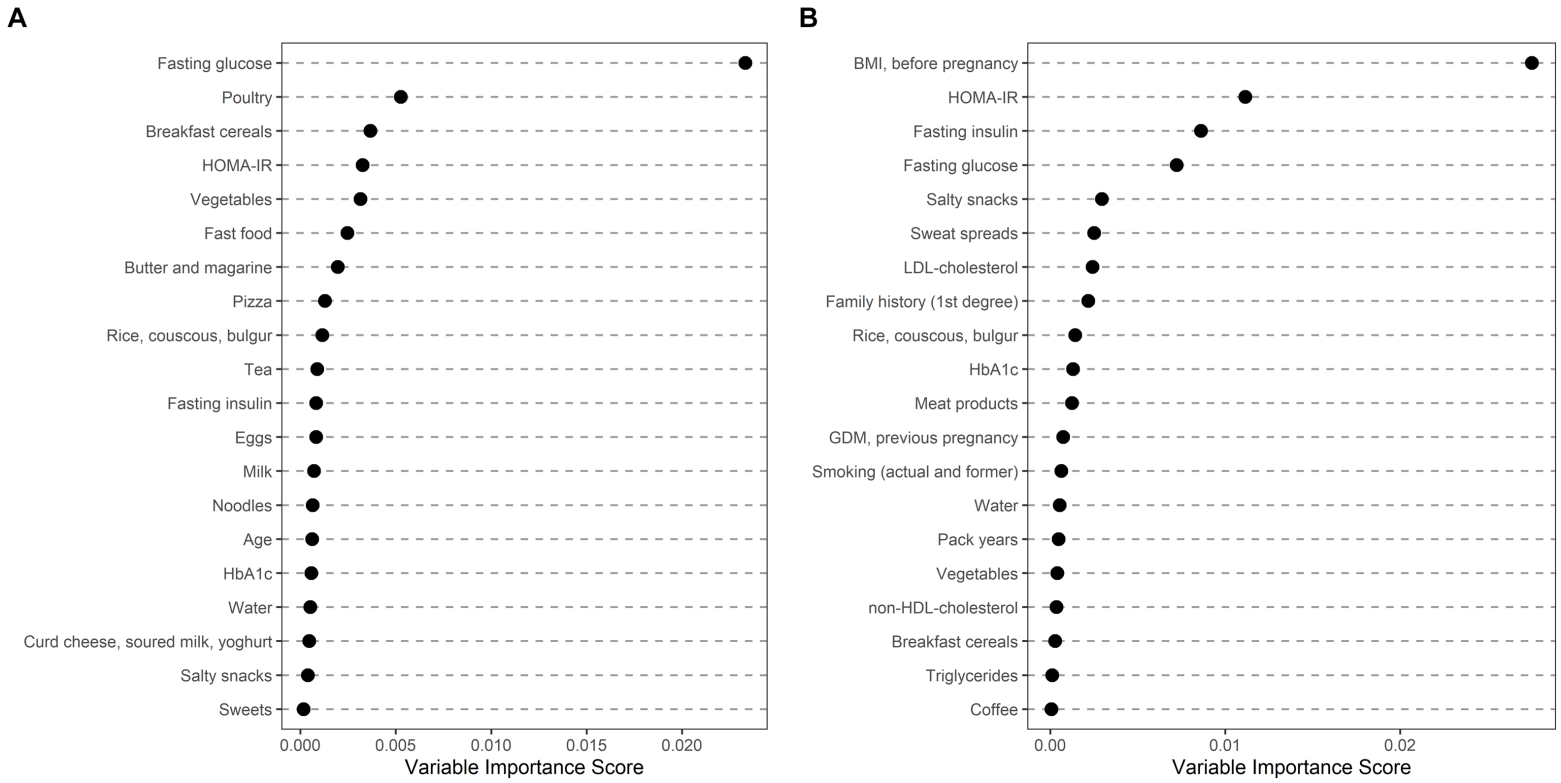
Ethnically stratified risk factors for GDM at early pregnancy showing variable importance scores for non-Caucasian mothers with Middle Eastern and Northern African **(A)** and Asian origin **(B)**. The first 20 variables with highest variable scores are shown.

Ethnically stratified variable importance metrics were calculated for 396 Caucasian (64 with GDM) and 114 non-Caucasian women (MENA: 63, 20 with GDM; ASIA: 46, 15 with GDM; SSA: 5, 1 with GDM) with complete information about baseline characteristics (as summarized in Table 2) and dietary habits (as summarized in Table 3). As visualized in Figure 1, fasting glucose as well as HOMA-IR achieved high variable importance scores in both Caucasian and non-Caucasian mothers. Pregestational BMI was a more important predictor in non-Caucasian mothers, whereas history of pregnancy with GDM was more important in Caucasian mothers. In general, dietary habits were of minor importance in both groups. The estimated out of bag error (as a measurement of prediction error of the random forest) was lower in Caucasian patients as compared to non-Caucasian mothers (16.7% vs. 26.1%). In a further analysis non-Caucasian mothers were stratified according to their regional origin and showed that fasting glucose was especially important in women from the MENA regions, whereas maternal pregestational BMI and insulin sensitivity status was more important in Asian mothers (Figure 2). Due to the restricted sample size women from the SSA region were excluded from this analysis.

## 4 Discussion

This study aimed to assess the role of ethnicity on the development of GDM in a prospectively assessed and well characterized multiethnic Central European cohort. We found that women with non-Caucasian ancestry, especially those with origin from the MENA and Asian countries have markedly increased risk as compared to Caucasian mothers. This is corroborated by results of some previous retrospective and register studies, suggesting a higher GDM incidence for specific ethnicities and minorities, such as South and East Asian, Indigenous Australian, African, Hispanics and Native Americans (24–28). Recently, Caputo et al. retrospectively analyzed data of 586 patients and found that despite being younger, GDM patients from “high migration pressure countries” required insulin treatment more often, what is also indicated for non-Caucasian mothers in our study (29). Likewise, a population based Norwegian register study identified substantially increased GDM incidence in immigrant women, whereby the risk for hyperglycemia increased in parallel to the length of residence in certain immigrant groups (9), suggesting, that the elevated risk in these mothers is not only attributable to a genetic predisposition (24).

In the present study, we also observed distinct differences in patient characteristics and clinical-metabolic features between Caucasian and non-Caucasian women. Thereby, non-Caucasian mothers had higher pregestational BMI, were more insulin resistant and showed an adverse glucometabolic risk profile with higher fasting glucose, and HbA1c already at start of gestation. In another study of reproductive-aged Austrian women, we previously found that women with origin from the MENA countries undergoing infertility treatment were more obese and, despite being younger as compared to Caucasian patients, showed impaired ovarian function, possibly explained by a higher incidence of Polycystic Ovarian Syndrome – a disease markedly triggered by impaired insulin sensitivity (30). This is comparable to our present study, indicating that especially women with origin from the MENA region had higher BMI as well as a higher degree of insulin resistance and consequently an increased risk for GDM development. Aside from the MENA population, we additionally observed an increased risk for GDM in mothers with Asian ancestry. This is in line with another recent register study, indicating that the risk of GDM is increased in mothers with South Asian and Chinese ethnicity, who had lower BMI as compared to the general Canadian population (11). Interestingly, Sharma et al. observed, that glucose metabolism remained markedly impaired after GDM pregnancy, in particular in Asian mothers, who showed impaired β-cell function, insulin action and clearance as compared to Nordic women (31).

Ethnically specific differences are often explained by different lifestyle habits, especially dietary patterns. Thereby, some authors suggested that “nutrition transition” towards an energy dense Western diet may promote the development of metabolic disorders and the requirement of glucose lowering medication in GDM patients (29, 32, 33). Dietary habits can also directly affect fetal development and growth and this effect can be possibly modulated by ethnicity. For example, Zulyniak et al. found that consumption of plant-based diet reduced infant birth weight in white Europeans, whereas it increased the risk for LGA infants in South Asians living in Canada (34). Another meta-analysis recently assessed the effect of healthy diet on GDM incidence and indicated significant associations between dietary patterns and GDM risk markedly in white European mothers, whereas no consistent evidence was observed in non-Caucasian populations, what may be explained by heterogenous use of dietary assessment tools (35). Differences in dietary patterns were also observed in our study, whereby non-Caucasian immigrants differed markedly from the Caucasian population. However, a detailed analysis of GDM risk factors indicated that dietary patterns had inferior variable importance scores as compared to other clinical-metabolic features, such as fasting glucose and maternal insulin resistance, showing consistently high predictive performance in Caucasian and non-Caucasian mothers. Of note, maternal pregestational BMI achieved notably higher importance for GDM prediction in Asian mothers. In line with our findings, Read et al. found that BMI increased the risk for GDM at far lower levels in South Asian and Chinese mothers, possibly indicating that limiting excess weight gain may be particular effective for GDM prevention in Asian mothers (11). This effect may be mediated by distinct metabolic profiles of Asian and white European women as recently suggested by another study (36).

Some advantages and limitations need to be addressed: Clinical and metabolic risk factors as well as dietary patterns were only assessed once at start of pregnancy, what may be seen as a limitation. However, the large sample size with a high proportion of non-Caucasian study participants and GDM cases is a clear advantage. In addition, the prospective character of the cohort study design allowed us to assess detailed information about ancestry (i.e., the parental country of origin) and allowed us to accurately determine patient’s ethnicity. Moreover, this is for our knowledge the first study including information of clinical features, metabolic parameters (such as early gestational insulin sensitivity) and dietary habits in a multiethnic cohort of pregnant women.

In summary, we identified distinct differences in dietary patterns and clinical metabolic features between Caucasian and non-Caucasian mothers, who showed a higher incidence of GDM and need of glucose lowering medications. GDM risk was highest in Asian mothers and those with origin from the MENA region. Fasting plasma glucose as well as maternal insulin resistance at start of pregnancy are important risk factors in both, Caucasian and non-Caucasian mothers, although, increased maternal BMI (even at lower levels) may be of particular importance in Asians. The information provided by our study is of clinical relevance to improve risk stratification and to provide “culturally appropriate care” (14) for non-Caucasian ethnicities indicating the need for further research in non-Caucasian populations.

## Data availability statement

The original contributions presented in the study are included in the article/Supplementary material, further inquiries can be directed to the corresponding author.

## Ethics statement

The studies involving humans were approved by the Ethics Committee of the Medical University of Vienna. The studies were conducted in accordance with the local legislation and institutional requirements. The participants provided their written informed consent to participate in this study.

## Author contributions

GK: Writing – original draft, Writing – review & editing, Data curation, Project administration. CM: Writing – original draft, Writing – review & editing. TL: Data curation, Writing – review & editing. DE: Writing – review & editing. VS: Writing – review & editing. MF: Writing – review & editing. BM: Writing – review & editing. VF: Writing – review & editing. SW: Writing – review & editing. WH: Writing – review & editing. AT: Formal analysis, Writing – review & editing. CG: Conceptualization, Data curation, Formal analysis, Visualization, Writing – original draft, Writing – review & editing.

## Footnotes

^1^ http://www.kimcl.at
