# Supplementary material for: The impact of regional origin on the incidence of gestational diabetes mellitus in a multiethnic European cohort
Source: Front Public Health. 2024 Jan 19;11:1286056. doi: 10.3389/fpubh.2023.1286056 (PMC10834617; doi:10.3389/fpubh.2023.1286056)
Supplement: Supplementary file 1 [file Table_1.docx]

**Supplemental Material, Table S1:** Early gestational characteristics in pregnant women according to the regional origin.

|  | **CAUC** | **MENA** | **SSA** | **ASIA** |
| --- | --- | --- | --- | --- |
| Age (years) | 32.0±5.9 | 31.0±5.8 | 30.9±5.7 | 31.2±5.2 |
| Parity (≥1) | 404 (55.3) | 144 (73.8)* | 43 (89.6)* | 93 (73.2)* |
| Parity (≥2) | 154 (21.1) | 82 (42.1)* | 29 (60.4)* | 49 (38.6)* |
| Parity (≥3) | 46 (6.3) | 39 (20.0)* | 14 (29.2)* | 25 (19.7)* |
| GDM, previous pregnancy | 67 (9.2) | 25 (12.8) | 8 (16.7) | 17 (13.4) |
| Family history (1st degree) | 164 (22.4) | 83 (42.6) | 5 (10.4) | 45 (35.4) |
| Assisted reproduction | 93 (12.7) | 19 (9.7) | 0 (0.0) | 7 (5.5) |
| Multiple pregnancy | 95 (13.0) | 24 (12.3) | 1 (2.1) | 7 (5.5) |
| Smoking status (actual smokers) | 128 (17.5) | 24 (12.3) | 0 (0.0) | 3 (2.4)* |
| Smoking status (former smokers) | 232 (31.7) | 32 (16.4)* | 0 (0.0) | 9 (7.1)* |
| Smoking status (actual and former smokers) | 360 (49.2) | 56 (28.7)* | 0 (0.0) | 12 (9.4)* |
| Pack years (a) | 0 (0-4) | 0 (0-0.4)* | 0 (0-0)* | 0 (0-0)* |
| Height (cm) | 166±6.6 | 163±6.1* | 165±6.6 | 160±5.9* |
| Weight, before pregnancy (kg) | 68±16 | 69±14 | 75±14* | 61±11* |
| BMI, before pregnancy (kg/m^2^) | 24.7±5.7 | 26.0±5.0* | 27.6±5.5* | 23.9±3.9 |
| RRS (mmHg) | 120±12 | 117±13* | 117±17 | 117±12* |
| RRD (mmHg) | 77±10 | 75±10* | 76±10 | 77±10 |
| Triglycerides, early pregnancy (mg/dl) | 119±47 | 123±46 | 84±31* | 126±47 |
| Total-cholesterol, early pregnancy (mg/dl) | 192±35 | 187±35 | 173±34* | 178±31* |
| LDL-cholesterol, early pregnancy (mg/dl) | 96±28 | 95±29 | 90±26 | 87±25* |
| HDL-cholesterol, early pregnancy (mg/dl) | 72±16 | 67±15* | 66±13 | 66±15* |
| non-HDL-Cholesterol (mg/dl) | 120±33 | 119±32 | 107±30* | 112±28* |
| FPG, early pregnancy (mg/dl) | 81.2±6.3 | 83.1±7.4* | 84.8±6.8* | 81.9±6.8 |
| HbA1c, early pregnancy (mmol/mol) | 30.7±3.2 | 31.2±3.6 | 32.4±4.1* | 32.2±3.3* |
| Fasting insulin, early pregnancy (µU/ml) | 7.7 (5.3-11.6) | 9.0 (6.3-12.1)* | 8.3 (6.3-13.5) | 9.1 (6.3-13.3) |
| HOMA-IR, early pregnancy (dimensionless) | 1.6 (1.0-2.4) | 1.8 (1.3-2.6)* | 1.7 (1.2-2.8) | 1.8 (1.2-2.7) |

Data are mean±SD or median (IQR) and count (%) for women with Caucasian (CAUC), Middle Eastern and Northern African (MENA), Sub-Saharan African (SSA) and Asian origin (ASIA). BMI, body mass index; RRS, systolic blood pressure; RRD, diastolic blood pressure; LDL, low density lipoprotein; HDL, high density lipoprotein; FPG, fasting plasma glucose; HbA1c, glycated hemoglobin A1c; HOMA-IR, homeostasis model assessment of insulin resistance

* p<0.05 vs. CAUC

**Supplemental Material, Table S2:** Dietary habits at early pregnancy according to the regional origin.

|  | **CAUC** | **MENA** | **SSA** | **ASIA** |
| --- | --- | --- | --- | --- |
| Milk (ml/d) | 100 (21-200) | 43 (4-189)* | 50 (9-200) | 100 (18-200) |
| Water (l/d) | 1.2 (0.9-4.8) | 1.2 (0.6-3.6)* | 0.9 (0.6-3.6) | 1.2 (0.6-3.6) |
| Non-alcoholic beverages (ml/d) | 200 (61-443) | 100 (27-300)* | 116 (35-200) | 112 (42-300)* |
| Coffee (ml/d) | 32 (0.0-150) | 6.0 (0.0-47)* | 0.0 (0.0-32) | 0.0 (0.0-5.4)* |
| Tea (ml/d) | 75 (8.0-182) | 225 (101-450)* | 75 (32-150) | 163 (32-602)* |
| Bread (g/d) | 82 (48-150) | 104 (59-202)* | 60 (23-100) | 88 (43-193) |
| Rice, couscous, bulgur (g/d) | 16 (7-32) | 32 (13-48)* | 75 (32-150)* | 32 (16-75)* |
| Noodles (g/d) | 27 (11-27) | 12 (6-27)* | 27 (13-27) | 11 (4-27)* |
| Potatoes (g/d) | 49 (26-84) | 40 (21-70) | 53 (21-93) | 38 (18-86) |
| Pizza (g/d) | 13 (6-31) | 13 (6-31) | 8 (1-16) | 8 (3-31)* |
| Breakfast cereals (g/d) | 3 (0-10) | 0 (0-4)* | 0 (0-5) | 1 (0-5)* |
| Legumes (g/d) | 7 (3-16) | 13 (3-32)* | 5 (1-13) | 11 (3-32) |
| Vegetables (g/d) | 88 (34-182) | 83 (38-165) | 38 (14-123) | 51 (14-125)* |
| Fruits (g/d) | 300 (130-450) | 155 (150-301) | 150 (75-236) | 300 (150-342) |
| Butter and magarine (g/d) | 4 (1-10) | 2 (0-10)* | 0 (0-3)* | 2 (0-8)* |
| Cheese (g/d) | 15 (5-30) | 30 (6-47) | 0 (0-3)* | 3 (0-7)* |
| Cream Cheese (g/d) | 3 (1-13) | 6 (1-15) | 0 (0-0)* | 1 (0-8)* |
| Curd cheese, soured milk, yoghurt (g/d) | 43 (9-100) | 43 (9-100) | 18 (1-43) | 43 (7-100) |
| Eggs (g/d) | 13 (5-26) | 30 (13-60)* | 13 (5-30) | 13 (11-47) |
| Meat (g/d) | 26 (5-26) | 11 (3-26)* | 26 (6-60) | 13 (3-30) |
| Meat products (g/d) | 12 (4-26) | 0 (0-4)* | 0 (0-3)* | 0 (0-5)* |
| Poultry (g/d) | 16 (13-32) | 13 (3-32)* | 19 (13-32) | 13 (1-32)* |
| Fish (g/d) | 8 (2-19) | 3 (0-9)* | 14 (7-22) | 3 (0-10)* |
| Fast Food (g/d) | 13 (0-25) | 10 (0-25) | 16 (0-27) | 10 (0-27) |
| Sweat spreads (g/d) | 3 (1-8) | 4 (1-14) | 2 (0-5) | 4 (1-10) |
| Sweets (g/d) | 38 (21-72) | 31 (14-74) | 18 (8-36)* | 22 (7-37)* |
| Salty snacks (g/d) | 4 (2-10) | 6 (2-13)* | 4 (1-9) | 3 (1-8) |

Data are mean±SD or median (IQR) and count (%) for women with Caucasian (CAUC), Middle Eastern and Northern African (MENA), Sub-Saharan African (SSA) and Asian origin (ASIA).

* p<0.05 vs. CAUC
